# Association between perioperative blood transfusion and length of hospital stay in patients with osteoporotic fractures

**DOI:** 10.1186/s12891-023-07101-6

**Published:** 2023-12-19

**Authors:** Si-ming Xu, Ke Lu, Xu-feng Yang, Yao-wei Ye, Min-zhe Xu, Qin Shi, Ya-qin Gong, Chong Li

**Affiliations:** 1 Department of Orthopedics, Gusu School, Nanjing Medical University, The First People’s Hospital of Kunshan, Suzhou, Jiangsu 215300 China; 2 Department of Orthopedics, Affiliated Kunshan Hospital of Jiangsu University, No. 566 East of Qianjin Road, Suzhou, Jiangsu 215300 China; 3 Department of Orthopedics, the First Affiliated Hospital of Soochow University, Orthopedic Institute of Soochow University, Suzhou, Jiangsu 215031 China; 4 Information Department, Affiliated Kunshan Hospital of Jiangsu University, Suzhou, Jiangsu 215300 China

**Keywords:** Perioperative blood transfusion, Length of hospital stay, Osteoporotic fracture

## Abstract

**Background:**

Few studies have examined the relationship between perioperative blood transfusion (PBT) and length of hospital stay (LOS) in patients with osteoporotic fractures. This research aims to study the association between PBT and LOS.

**Methods:**

This is a retrospective cross-sectional study from the Affiliated Kunshan Hospital of Jiangsu University, Suzhou, China, involving 2357 osteoporotic fractures (OPF) patients who received surgical treatment during hospitalization from January 2017 and March 2022. Multiple linear regression was used to analyze the relationship between PBT and LOS. In the analysis, PBT volume was the dependent variable, whereas LOS was the independent variable. Simultaneously, age, gender, body mass index, hemoglobin, primary diagnosis, American Society of Anesthesiologists, creatinine (Cr), anesthesia, surgical method, and Charlson comorbidity index were included as covariates. The generalized additive model was then used to study nonlinear associations. Two piecewise linear regression exemplary evaluated the inception results for smoothing the curve.

**Results:**

Our results proved that PBT was positively correlated with LOS in the fully adjusted model (β, 0.21; 95% CI, 0.04 to 0.37; *P* < 0.0001). Furthermore, a “U-shape” nonlinear relationship existed between PBT and LOS. When the concentration of PBT was between 0 and 1.5 units, it was manifested as a negative correlation between PBT and LOS. However, there was a positive association between PBT and LOS when PBT levels exceeded 1.5 units.

**Conclusions:**

This study demonstrated that PBT and LOS in the OPF population were independent with a nonlinear relationship. These results suggest that PBT may be protective for patients with long LOS. If these findings are confirmed, the LOS in OPF patients can be regulated through appropriate perioperative blood transfusion.

## Introduction

Osteoporosis (OP) is a skeletal condition characterized by reduced bone mass and deteriorated bone microarchitecture, leading to an increased risk of bone fractures [1]. It is a significant public health concern, especially in China, where the estimated age-standardized prevalence of OP at the spine or hip among individuals aged 50 years and above was 6.46% in males and 29.13% in females in 2019 [2]. These numbers indicate a substantial burden of osteoporosis in the population, with an estimated 10.9 million Chinese males and 49.3 million females projected to have OP. Several risk factors have been identified for OP, including lifestyle choices, dietary habits, comorbidities, medications, and genetic predisposition [2, 3].

Diagnosis of OP is primarily based on measurements of bone mineral density (BMD) [1] However, the clinical management of osteoporosis goes beyond diagnosis and extends to the prevention and treatment of osteoporotic fractures (OFs). OFs are common among individuals with OP, particularly in those over the age of 65, and often require surgical intervention, especially in the case of hip fractures [4]. Surgical procedures for OFs can result in substantial blood loss, leading to anemia, which necessitates perioperative blood transfusions (PBT) [4].

While PBT is a common practice in the management of OFs, there are potential hazards associated with blood transfusion. These include the transmission of infectious diseases and the emerging evidence suggesting an association between blood transfusion and adverse outcomes [5]. Hence, it is essential to investigate the relationship between PBT volumes and clinical outcomes, such as length of hospital stay (LOS), in individuals with osteoporotic fractures.

Length of hospital stay (LOS) is a crucial metric for evaluating the effectiveness of care provided to individuals with OFs and allows for benchmarking [6]. Prolonged LOS in trauma patients has been associated with an increased risk of in-hospital complications, including urinary tract infections, pneumonia, sepsis, pressure ulcers, delirium, and deep vein thrombosis [7]. Moreover, extended LOS with in-patient complications is associated with increased hospitalization costs [8]. Identifying individuals at an early stage who are at high risk for prolonged LOS is essential for providing efficient and prompt interventions. Early detection can reduce hospitalization expenses, alleviate the burden on medical resources, and reduce the incidence of in-hospital complications [9].

The aim of this study is to investigate the independent association between PBT volumes and LOS in Chinese patients with osteoporotic fractures. By elucidating the relationship between blood transfusion and clinical outcomes, this research can contribute to the understanding of the optimal management strategies for individuals with OP and OFs. The findings of this study have potential clinical significance, providing insights for healthcare providers in improving patient care, optimizing resource allocation, and reducing the burden of complications associated with prolonged hospital stays in this patient population.

## Materials and methods

### Study design and patients’ data

**Fig. 1:**
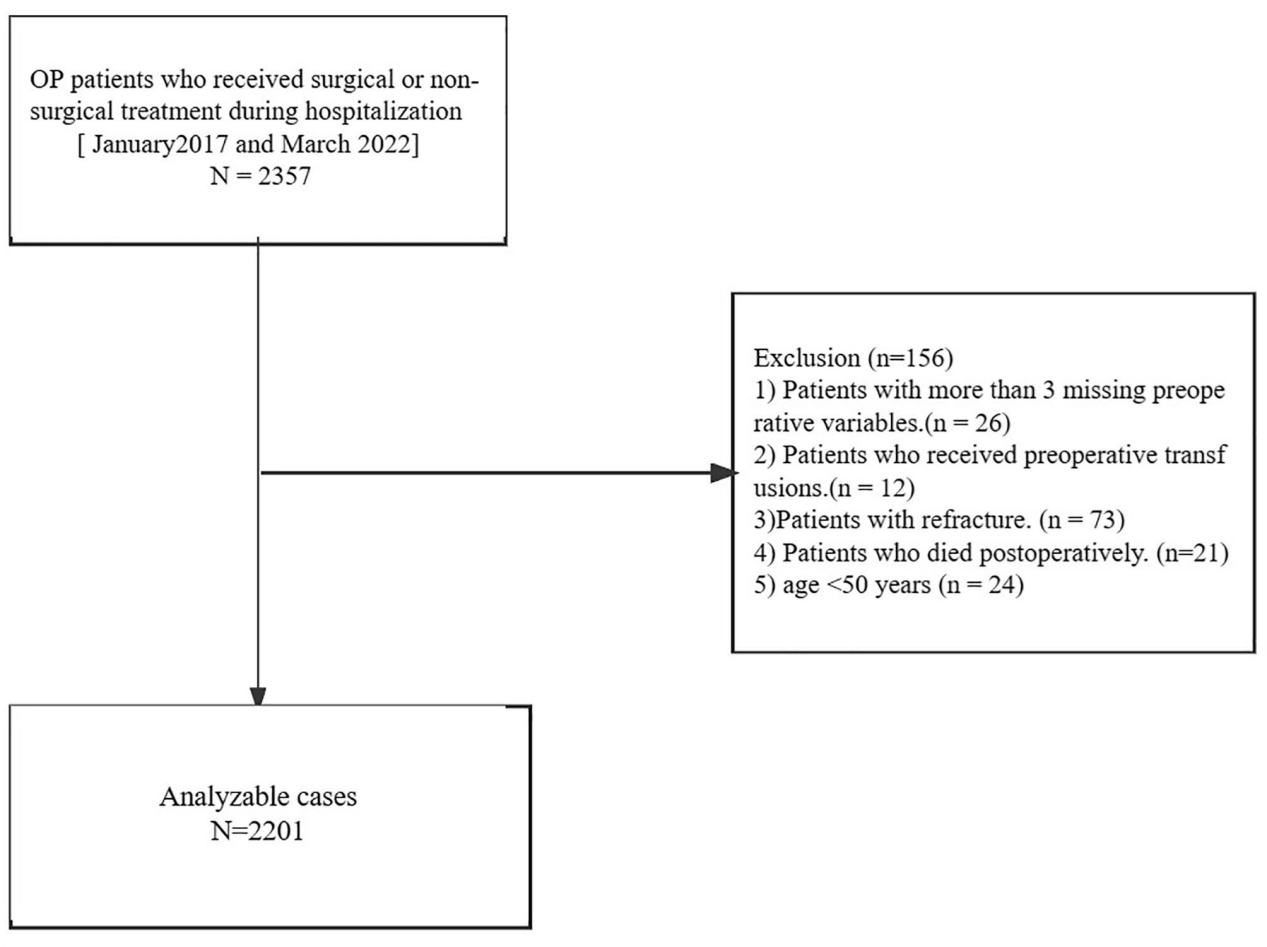
A schematic of our study design

This study employed a retrospective investigation design to analyze the relationship between perioperative blood transfusion (PBT) volumes and length of hospital stay (LOS) in Chinese patients with osteoporotic fractures (OFs). The study period spanned from January 2017 to March 2022. The patient data were obtained from the electronic medical records of the Affiliated Kunshan Hospital of Jiangsu University, Suzhou, China. The study included a total of 2357 patients diagnosed with osteoporosis (OP) who received medical blood checks during their hospitalization. To establish the diagnosis of OP, the following inclusion criteria were applied: (1) Presence of bone instability and fractures without any other metabolic bone diseases, along with physiological bone mineral density (BMD) measured by T-score. (2) Confirmation of OP based on a T-score of -2.5 or less, even in the absence of a predominant bone fracture [1]. The exclusion criteria are as follows: (1) patients with more than three missing preoperative variables (n = 26), or (2) patients with preoperative transfusion (n = 12); (3) patients with refracture (n = 73); (4) patients with postoperative death (n = 21); (5) age < 50 years (n = 24). After applying the inclusion criteria, 2201 patients were acquired for the study. Figure 1 depicts patients’ data and medical history. This study was approved by the Ethics Committee at the Affiliated Kunshan Hospital of Jiangsu University, Suzhou, China (approval No. 2020-03-046-K01) and was compliant with the Declaration of Helsinki. The patients’ identity was hidden for an unbiased investigation. All patients signed a written consent form.

### Dependent variables

The primary outcome was prolonged LOS, defined as more than 13 days. The average LOS refers to the average length of stay for each patient within a certain period. It is a relatively rigid comprehensive index to evaluate the medical benefit and efficiency, medical quality, and technical level. This cut-off was selected as it represents > 75th centile LOS of the entire sample. Other studies have used the 75th centile to define prolonged LOS [2].

### Exposure variables

The exposure variable in the present study was a perioperative blood transfusion. The perioperative period is the time between when the patient and the doctor decide on surgical therapy and 28 days after surgery (basic recovery). Perioperative blood transfusion refers to the administration of blood or blood components to a patient during the perioperative period, which includes the preoperative, intraoperative, and postoperative phases of surgical care. It involves the transfusion of blood products, such as red blood cells, platelets, and plasma, to replenish or replace blood loss or correct anemia that may occur as a result of surgery or other medical interventions [3].

### Covariates

Age, gender, BMI (body mass index), This research classified the patients into four groups based on the estimated BMI (kg/m^2^): (1) BMI ≥ 18.5, where < 25 is considered normal; (2) BMI < 30, where ≥ 25 is considered overweight; (3) BMI < 35, where ≥ 30 regarded fatness; (4) BMI ≥ 35 is considered obesity. hemoglobin, primary diagnosis, ASA (American Society of Anesthesiologists), Cr (creatinine), anesthesia, surgical method, and CCI (Charlson comorbidity index) were all served as covariates. All clinical variables were quantified within three days of hospital admittance.

### Statistics

All the results are presented as mean ± standard deviation (SD), as a median (Q1 Q3), and frequency (%) for constant and categorical variables, respectively. Pearson’s chi-square or Fisher’s exact tests were used for univariate data quantitating absolute values. Continuous variables with standard and non-normally distributed data were analyzed using the t-test and the Mann-Whitney U test, respectively. The univariate logistic regression data quantitation assessed the relationship between PBT and LOS.

Controlling the influence of covariances, the generalized estimating Eq. [4] studied the independent relations between perioperative blood transfusion and LOS. Unadjusted (basic), negligibly accustomed (Model I), or fully adjusted (Model II) models were used to quantify the data. The evaluation of variance inflation factor (VIF) data allowed for the adjustment of covariances based on the following criteria: (1) the covariate was included in the crude model or detached from the full one, whereas the similar odds ratio (OR) was altered by at least 10%; (2) the covariate from criterion 1 had *P*-value < 0.1 in the univariate model [5]. Model II was developed using criteria 1 in terms of fully adjusted models.

Our data were found to have nonlinear correlations using the generalized additive model (GAM). The two-piecewise linear regression model sets the threshold for line smoothing. In the case of an apparent ratio in the smoothing curve, the recursive method was used to spontaneously evaluate the turning point [6]. Furthermore, this research performed subgroup analyses and estimated their robustness and potential variations, stratifying different covariates. Finally, the modifications and interactions of the subgroups were analyzed using the likelihood ratio test (LRT).

All statistical analyses were performed using the Empower Stats (www.empowerstats.com, X&Y Solutions, Inc., Boston, MA, USA). The R software version 3.6.3 was also applied (http://www.r-project.org). *P*-values less than 0.05 were considered statistically significant.

## Results

### Features of study contributors and univariate analysis

A population-descriptive analysis was performed to define the osteoporosis population included in the study. For 2341 patients, LOS was delimited by 75% of the study population and divided into ≤ 13 and > 13, among which 1720 had LOS ≤ 13, and 481 had LOS > 13. In people with LOS ≤ 13, 71.92% (n = 1237) were female, and 53.95% (928) were ≥ 70 years old and passed the inclusion criteria and enrolled in this retrospective investigation. Table 1 presents the medical data. And in people with LOS > 13, 63.83%% (n = 307) were female, and 68.81% (n = 331) were ≥ 70 years old. In people with LOS ≤ 13, the BMI for the study participants was normal at 76.10% (n = 1309). For patients with LOS > 13, the BMI for the study participants was normal at 80.46% (n = 387). This study divided perioperative transfusions into three categories: (1) those who did not undergo perioperative transfusions; (2) those who had one unit transfusion; (3) those who had ≥ 2 units transfusion. There were 92.79% (n = 1596) of patients with LOS ≤ 13 who did not receive a blood transfusion, 2.15% (n = 37) who received one unit, and 5.06% (n = 87) who received more than two units. There were 88.36% (n = 425) of patients with LOS > 13 who did not receive a blood transfusion, 2.08% (n = 10) who received one unit, and 9.56% (n = 46) who received more than two units. This study diagnosed patients in three categories: (1) vertebral fracture, (2) hip fracture, and (3) limb fracture. Vertebral fractures accounted for 49.83% (n = 857) of those with LOS ≤ 13, hip fractures 31.63% (n = 544), and limb fractures 18.55% (n = 319). For those with LOS > 13, vertebral fractures accounted for 17.46% (n = 84), hip fractures 75.68% (n = 364), and limb fractures 6.86% (n = 33). There are four levels for the ASA (American Society of Anesthesiologists) score: 1, 2, 3, and 4. For those patients with LOS ≤ 13, the ratios are 7.79% (n = 134), 65.76% (n = 1131), 26.22% (n = 451), and 0.23% (n = 4). Further, the ratios are 3.33% (n = 16), 57.17% (n = 275), 38.46% (n = 185), and 1.04% (n = 5) for patients with LOS > 13. Hemoglobin concentration was graded into four categories: 1) ≥ 130 g/L, 2) < 130 g/L, ≥ 120 g/L, 3) < 120 g/L, ≥ 110 g/L, and 4) < 110 g/L. The surgical methods were divided into 12 types: (1) open reduction and plate internal fixation of radius fracture, (2) external fixation of radius fracture, (3) femoral head replacement, (4) total hip replacement, (5) closed reduction and internal fixation of the femur, (6) open reduction and internal fixation of the femur, (7) percutaneous kyphoplasty, (8) vertebroplasty, (9) internal fixation of the vertebra, and open reduction and internal fixation of the femur, (10) lumbar posterior column fusion, posterior approach, 11) closed reduction and intramedullary nailing fixation of humeral fractures, and 12) open reduction and internal fixation of humeral fractures. Anesthesia is divided into five types: (1) General anesthesia, (2) Local anesthesia, (3) Spinal anesthesia, (4) Continuous epidural anesthesia, and (5) Brachial plexus anesthesia. The CCI (Charlson comorbidity index) score is divided into six levels.

**Table 1:** Characteristics of study participants

Variable	LOS ≤ 13 N = 1720	LOS > 13 N = 481	*p*-value
Cr mean (SD)^a^	66.93 (31.64)	65.11 (45.13)	0.01
Gender n (%)			< 0.001
Female	1237 (71.92%)	307 (63.83%)	
Male	483 (28.08%)	174 (36.17%)	
AGE n (%)			< 0.001
< 60	248 (14.42%)	47 (9.77%)	
≥ 60, < 65	218 (12.67%)	36 (7.48%)	
≥ 65, < 70	326 (18.95%)	67 (13.93%)	
≥ 70	928 (53.95%)	331 (68.81%)	
BMI (kg/m2) n (%)			0.22
≥ 35	3 (0.17%)	1 (0.21%)	
< 35, ≥ 30	29 (1.69%)	8 (1.66%)	
< 30, ≥ 25	379 (22.03%)	85 (17.67%)	
≥ 18.5, < 25	1309 (76.10%)	387 (80.46%)	
Perioperative blood transfusion n (%)			0.002
None	1596 (92.79%)	425 (88.36%)	
One unit	37 (2.15%)	10 (2.08%)	
≥ 2 units	87 (5.06%)	46 (9.56%)	
Primary diagnosis n (%)			< 0.001
Vertebral fracture	857 (49.83%)	84 (17.46%)	
Hip fracture	544 (31.63%)	364 (75.68%)	
Limb fracture	319 (18.55%)	33 (6.86%)	
ASA n (%)			< 0.001
1	134 (7.79%)	16 (3.33%)	
2	1131 (65.76%)	275 (57.17%)	
3	451 (26.22%)	185 (38.46%)	
4	4 (0.23%)	5 (1.04%)	
Hemoglobin (g/L) n (%)			0.60
≥ 130	740 (43.68%)	221 (47.02%)	
< 130, ≥ 120	388 (22.90%)	101 (21.49%)	
< 120, ≥ 110	280 (16.53%)	70 (14.89%)	
< 110	286 (16.88%)	78 (16.60%)	
surgical method n (%)			< 0.001
Open reduction plate internal fixation for radial fracture	226 (13.14%)	22 (4.57%)	
External radial fixation	4 (0.23%)	0 (0.00%)	
Femoral head replacement	110 (6.40%)	94 (19.54%)	
Total hip replacement	27 (1.57%)	30 (6.24%)	
Closed reduction and internal fixation of the femur	356 (20.70%)	197 (40.96%)	
Open reduction and internal fixation of the femur	45 (2.62%)	40 (8.32%)	
Percutaneous kyphoplasty	706 (41.05%)	43 (8.94%)	
Spondyloplasty	96 (5.58%)	5 (1.04%)	
Vertebrae internal fixation	50 (2.91%)	34 (7.07%)	
Lumbar posterior column fusion, posterior approach	5 (0.29%)	3 (0.62%)	
Closed reduction intramedullary pin fixation of humerus fracture	9 (0.52%)	2 (0.42%)	
Open reduction of humerus fracture with internal fixation	86 (5.00%)	11 (2.29%)	
Anesthesia n (%)			< 0.001
General anesthesia	347 (20.17%)	112 (23.28%)	
Local anesthesia	571 (33.20%)	24 (4.99%)	
Spinal anesthesia	473 (27.50%)	302 (62.79%)	
Continuous epidural anesthesia	15 (0.87%)	11 (2.29%)	
Brachial plexus anesthesia	314 (18.26%)	32 (6.65%)	
CCI n (%)			< 0.001
0	1570 (91.28%)	371 (77.13%)	
1	118 (6.86%)	79 (16.42%)	
2	23 (1.34%)	22 (4.57%)	
3	6 (0.35%)	5 (1.04%)	
4	2 (0.12%)	3 (0.62%)	
5	1 (0.06%)	0 (0.00%)	
7	0 (0.00%)	1 (0.21%)	

^a^For continuous variables

Abbreviations: SD, standard deviation; Cr, creatinine; BMI, body mass index; ASA, American Society of Anesthesiologists; CCI, Charlson comorbidity index

### Univariate analysis for LOS

Table 2 depicts the results of the univariate logistic regression analysis. This research has shown that the perioperative blood transfusion was positively related to the LOS (β, 0.7; 95% CI [confidence interval], 0.5, 0.8; *P* < 0.001).

**Table 2:** Univariate analysis for LOS

Variable	β^a^(95%CI)	*p*-value
Perioperative blood transfusion	0.7 (0.5, 0.8)	< 0.001
Cr	-0.0 (-0.0, 0.0)	0.268
Gender		
Female	Reference	
Male	1.4 (0.9, 2.0)	< 0.001
AGE		
< 60	Reference	
≥ 60, < 65	-0.2 (-1.2, 0.7)	0.621
≥ 65, < 70	0.0 (-0.8, 0.9)	0.921
≥ 70	1.4 (0.6, 2.1)	< 0.001
BMI (kg/m2)		
≥ 35	Reference	
< 35, ≥ 30	-3.3 (-9.5, 3.0)	0.306
< 30, ≥ 25	-2.6 (-8.5, 3.4)	0.396
≥ 18.5, < 25	-2.2 (-8.1, 3.8)	0.477
Perioperative blood transfusion		
None	Reference	
One unit	0.7 (-1.0, 2.4)	0.403
≥ 2 units	3.3 (2.3, 4.4)	< 0.001
Primary diagnosis		
Vertebral fracture	Reference	
Hip fracture	1.3 (0.6, 1.9)	< 0.001
Limb fracture	5.2 (4.7, 5.7)	< 0.001
ASA		
1	Reference	
2	0.9 (-0.0, 1.9)	0.060
3	2.8 (1.7, 3.8)	< 0.001
4	11.2 (7.3, 15.0)	< 0.001
Hemoglobin (g/L)		
≥ 130	Reference	
< 130, ≥ 120	-0.5 (-1.1, 0.1)	0.118
< 120, ≥ 110	-0.4 (-1.2, 0.3)	0.218
< 110	-0.5 (-1.2, 0.2)	0.180
surgical method		
Open reduction plate internal fixation for radial fracture	Reference	
External radial fixation	-1.2 (-6.5, 4.1)	0.664
Femoral head replacement	5.5 (4.5, 6.4)	< 0.001
Total hip replacement	5.1 (3.6, 6.6)	< 0.001
Closed reduction and internal fixation of the femur	3.4 (2.7, 4.2)	< 0.001
Open reduction and internal fixation of the femur	5.4 (4.2, 6.7)	< 0.001
Percutaneous kyphoplasty	-1.7 (-2.5, -1.0)	< 0.001
Spondyloplasty	-0.6 (-1.7, 0.6)	0.351
Vertebrae internal fixation	4.8 (3.5, 6.2)	< 0.001
Lumbar posterior column fusion, posterior approach	5.1 (1.5, 8.7)	0.005
Closed reduction intramedullary pin fixation of humerus fracture	2.0 (-1.2, 5.3)	0.216
Open reduction of humerus fracture with internal fixation	0.8 (-0.5, 2.0)	0.230
Anesthesia		
General anesthesia	Reference	
Local anesthesia	-3.9 (-4.5, -3.2)	< 0.001
Spinal anesthesia	2.3 (1.7, 2.9)	< 0.001
Continuous epidural anesthesia	3.1 (0.9, 5.3)	0.005
Brachial plexus anesthesia	-1.6 (-2.3, -0.8)	< 0.001
CCI		
0	Reference	
1	3.4 (2.6, 4.2)	< 0.001
2	5.0 (3.4, 6.7)	< 0.001
3	6.4 (3.2, 9.6)	< 0.001
4	11.7 (5.9, 17.5)	< 0.001
5	-2.5 (-14.1, 9.1)	0.671
7	14.5 (2.9, 26.1)	0.014

^a^The dependent variable was LOS and β, which results from univariate analysis for LOS level

Abbreviations: SD, standard deviation; CI, confidence interval; Cr, creatinine; BMI, body mass index; ASA, American Society of Anesthesiologists; CCI, Charlson comorbidity index

In addition to the relationship between PBT and LOS, this study also examined the impact of various demographic and clinical factors on LOS in patients with osteoporotic fractures (OFs). The analysis showed that males had a 140% longer LOS compared to females (*P* < 0.00001), and patients aged ≥ 70 years had a 140% longer LOS than those aged < 60 years (*P* < 0.00001). Patients who received more than two units of blood transfusion had a 330% longer hospital stay than those who did not receive a blood transfusion (*P* < 0.00001). Patients with hip and limb fractures had 130% and 520% longer LOS, respectively than those with vertebral fractures (*P* < 0.00001). Moreover, patients with American Society of Anesthesiologists (ASA) scores of 3 and 4 had 280% and 1120% longer LOS, respectively, than those with an ASA score of 1. Patients with a Charlson Comorbidity Index (CCI) score of 1, 2, 3, and 4 had 340%, 500%, 640%, and 1170% longer LOS, respectively than those with a CCI score of 0. These findings suggest that several demographic and clinical factors significantly impact LOS in patients with OFs and should be considered while managing these patients.

### Independent relation between the perioperative blood transfusion and LOS of patients

In evaluating the relationship between perioperative PBT and LOS in patients with osteoporotic fractures (OFs), multiple regression equations were utilized to control for confounding factors. Table 3 summarizes the results of the multivariate linear regression analysis, which examined the independent association between PBT and LOS. The crude unadjusted model, as well as Model I and Model II, were used to adjust for age, BMI, gender, hemoglobin, primary diagnosis, American Society of Anesthesiologists (ASA) score, creatinine (Cr), anesthesia, surgical method, and Charlson Comorbidity Index (CCI). The analysis revealed a significant positive relationship between PBT and LOS in all models, including the crude model (β, 0.66; 95% CI, 0.48 to 0.85; *P* < 0.0001), Model I (β, 0.58; 95% CI, 0.39 to 0.76; *P* < 0.0001), and Model II (β, 0.21; 95% CI, 0.04 to 0.37; *P* < 0.0001). The results indicate that an increase in PBT by one unit led to a 0.58-day (β, 0.58; 95% CI, 0.48 to 0.85; *P* < 0.0001) increase in LOS in Model I or a 0.21-day (β, 0.21; 95% CI, 0.04 to 0.37; *P* < 0.0001) increase in LOS in Model II. These findings suggest that PBT, even after controlling for various confounding factors, significantly predicts prolonged LOS in patients with OFs.

**Table 3:** Independent relationship between Perioperative blood transfusion and LOS

	Crude Model^a^		Model I^b^		Model II^c^	
	β (95%CI)	*P*-value	β (95%CI)	*P*-value	β (95%CI)	*P*-value
Perioperative blood transfusion	0.66 (0.48, 0.85)	<0.0001	0.58 (0.39, 0.76)	<0.0001	0.21 (0.04, 0.37)	0.0170

^a^No adjustment

^b^Adjusted for AGE; BMI; Gender; Hemoglobin

^c^Adjusted for Model I plus Primary diagnosis; ASA; Cr; Anesthesia; surgical method; CCI.

Abbreviations: CI, confidence interval; LOS, length of hospital stay; Cr, creatinine; BMI, body mass index; ASA, American Society of Anesthesiologists; CCI, Charlson comorbidity index

### Threshold analysis and spline smoothing plot

**Fig. 2:**
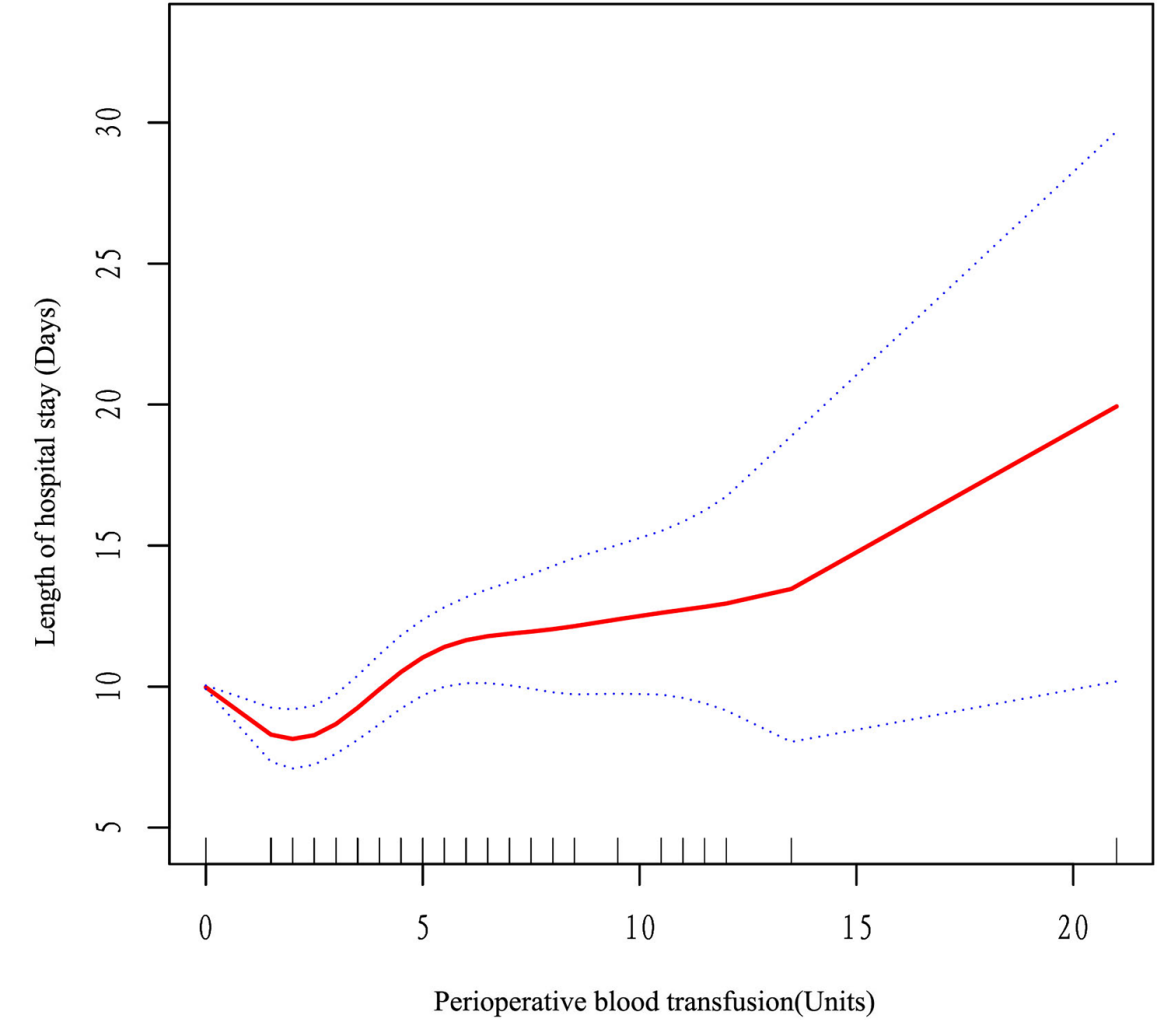
The adjusted smoothed curves of PBT and LOS. A threshold, nonlinear correlation between PBT and LOS, as evidenced by our generalized additive model. The red curve (middle) refers to a predicted value, and the blue curves (both sides) refer to the 95% CIs. Adjustment AGE; BMI; Gender; Hemoglobin; Primary diagnosis; ASA; Cr; Anesthesia; surgical method; CCI. The turning point (K) of the curve in Model II was 1.5 units

This study aimed to investigate a nonlinear relationship between PBT and LOS in patients with osteoporotic fractures (OP). In achieving this objective, a threshold effect analysis was conducted. Table 4 depicts the results of the analysis, showing that the fully adjusted Model II revealed a nonlinear relationship between PBT and LOS, as indicated by a *p*-value (< 0.05) for the Likelihood-Ratio Test. A two-piecewise linear regression model was used to identify the turning point (K) of the PBT at 1.5 units, resulting in a flattened curve. The findings demonstrated a significant negative association between PBT and LOS when the PBT range was 0 to 1.5 units (β, -1.4; 95% CI, -2.1 to -0.7; *P* < 0.001). However, a positive association was observed between PBT and LOS when the PBT range was > 1.5 units (β, 0.6; 95% CI, 0.4 to 0.9; *P* < 0.001). Figure 2 illustrates the relationship between PBT and LOS. These results suggest that excessive PBT volumes may increase LOS, whereas limited PBT volumes may decrease LOS. Thus, PBT should be carefully monitored and administered to minimize the risk of adverse outcomes and hospitalization costs.

**Table 4:** Threshold effect analysis examining the relationship between Perioperative blood transfusion and LOS

	Model II^a^	
	LOS	
	*β* (95%CI)	*P*-value
Model A^b^		
One line slope	0.2 (0.0, 0.3)	0.035
Model B^c^		
Perioperative blood transfusion turning point (K)	1.5	
<K	-1.4 (-2.1, -0.7)	< 0.001
>K	0.6 (0.4, 0.9)	< 0.001
Slope 2 – Slope 1	2.0 (1.1, 2.9)	< 0.001
LRT^d^	< 0.001	

^a^Adjusted for AGE; BMI; Gender; Hemoglobin; Primary diagnosis; ASA; Cr; Anesthesia; surgical method; CCI.

^b^Linear analysis, *P*-value < 0.05 indicates a linear relationship

^c^Non-linear analysis

^d^*P*-value < 0.05 means Model B is significantly different from Model A, which indicates a non-linear relationship

Abbreviations: CI, confidence interval; OR, odds ratio; LOS, length of hospital stay; LRT, logarithmic likelihood ratio test

The findings of this study have significant consequences for clinical practice and patient outcomes. Excessive use of PBT in patients with OFs should be carefully assessed, as it may raise the risk of adverse effects, longer hospital stays, and increased healthcare expenses. In contrast, limited PBT volumes may result in a shorter hospital stay and improved patient outcomes. Therefore, healthcare professionals must monitor PBT levels and make informed decisions about its administration. Moreover, future research should explore the impact of other factors, such as preoperative anemia and transfusion thresholds, on the relationship between PBT and LOS in patients with OFs. Such research could provide valuable insights for optimizing the use of PBT and improving patient outcomes.

## Discussion

This study represents the first epidemiological investigation in China to examine the independent association between PBT and LOS in individuals with osteoporotic fractures (OFs). The results of our analysis indicate a positive correlation between PBT and LOS in OFs patients. Furthermore, our study identified a nonlinear relationship between PBT and LOS, with a breakpoint of 1.5 units for PBT concentrations. These findings contribute to the existing literature on the impact of PBT on LOS in OFs patients, emphasizing the importance of carefully considering PBT administration to optimize patient outcomes and reduce healthcare costs.

The mechanism by which perioperative blood transfusion may shorten the mean length of hospital stay is a complex and multifactorial process. There are several potential mechanisms that can contribute to this effect, although it is important to note that the specific impact may vary depending on individual patient characteristics, the underlying condition, and the context of the surgical procedure. Here are some possible mechanisms: 1). Improved Oxygen Delivery: Perioperative blood transfusion can increase oxygen-carrying capacity by replenishing red blood cells and improving oxygen delivery to tissues. This may enhance tissue healing, reduce the risk of complications, and promote faster recovery, resulting in a shorter hospital stay. 2). Correction of Anemia: Anemia, characterized by a low hemoglobin level, is associated with decreased oxygen-carrying capacity and tissue hypoxia. Blood transfusion can correct anemia, which may help optimize tissue oxygenation, improve organ function, and potentially shorten the length of hospitalization. 3). Surgical Stress Response Reduction: Surgery and perioperative stress can lead to an inflammatory response and immune activation. Anemia exacerbates this stress response. Blood transfusion may mitigate the inflammatory response, modulate the immune system, and potentially reduce the duration of hospital stay. 4). Management of Complications: In some cases, perioperative blood transfusion may be required to manage and treat complications such as bleeding or hemorrhage. By promptly addressing these complications, transfusions may prevent further complications, expedite recovery, and contribute to a shorter hospital stay. [7–9].

It is important to note that while perioperative blood transfusion can have potential benefits, it is not without risks. Transfusion-related complications and adverse events can occur, including transfusion reactions, infections, and immunological effects. The decision to transfuse should always be based on careful assessment of the patient’s clinical condition, individualized risk-benefit analysis, and adherence to transfusion guidelines. [10].

Overall, the mechanism by which perioperative blood transfusion may shorten the mean length of hospital stay is likely multifactorial, involving improved oxygen delivery, correction of anemia, modulation of the surgical stress response, and effective management of complications. However, further research is needed to fully elucidate the underlying mechanisms and to better understand the impact of transfusion on patient outcomes.

Perioperative blood transfusion (PBT) is a widely accepted treatment for pre-existing anemia and blood loss, as well as to improve tissue oxygenation in patients with hip fractures. Studies suggest that PBT may improve healing during and after surgery, resulting in better functional outcomes and higher hemoglobin concentrations in patients with hip fractures [11]. Red blood cell transfusion carries the same risks as any other blood component. Incorrectly transfused blood components (due to mistakes in the processing or delivery routes) induce acute hemolytic responses, transfusion-transmitted infections, various forms of hemolytic reactions, respiratory problems, and allergy reactions. A 2-unit red blood cell transfusion has a volume of more than 500 mL, which might produce transfusion-associated circulatory overload in elderly patients who may be unable to tolerate even mild fluid infusions due to concurrent illnesses such as heart disease [10]. This results in an increase in LOS.

UK transfusion facilities distribute over two million red blood cell units annually, making red blood cell transfusion a commonly utilized therapeutic intervention [10]. Despite clinical study evidence showing no obvious benefit, red blood cell transfusions are often administered to stable, non-bleeding individuals [12]. Transfusions of red blood cells are frequently given together with hip fracture surgery. Moreover, postoperative functional recovery, mobility, and quality of life are significant outcomes in these patients [13, 14]. The LOS used in this study may indirectly indicate patients’ postoperative functional recovery, mobility, and quality of life. Notably, this research found a nonlinear relationship between PBT and LOS. When the concentration of PBT was less than 1.5 units, they were negatively correlated; however, when it was above 1.5 units, there was a positive correlation. These data are unique as they are not reported in previous studies.

Wendy F Bower suggested that perioperative blood transfusion increases LOS and causes complications for several days after surgery in non-cardiac surgical patients [15]. According to Xiao Cai et al., LOS decreases with increasing hemoglobin values; hence preoperative anemia should be corrected [16], contracting the present results. This contradiction could be inconsistencies in the study population, which did not include OFs patients from China. Although the age group was similar to our cohort of patients, blood transfusions are frequently necessary for individuals with osteoporotic fractures, resulting in different outcomes.

## Study strengths and limitations

This report is the first epidemic Chinese investigation that studied the independent association between the PBT and LOS of OP individuals. Our study might have some direct implications for clinical practice. First, patients with osteoporotic fractures benefitted from fewer than 1.5 units of PBT with shorter LOS. Moreover, this study showed that individuals with osteoporotic fractures had longer LOS when their PBT was higher. Second, perioperative blood transfusion can cause LOS when PBT does not exceed 1.5 units, lowering the risk of postoperative complications, improving preoperative anemia status, and replenishing blood volume in some surgical patients [16]. Finally, this research found helpful information for formulating relevant medical guidelines from an evidence-based perspective.

The study has certain drawbacks. First, this research found a link between PBT and LOS, but we could not prove the underlying connection between PBT and LOS. Further, some significant biological parameters, such as serum ferritin and mean corpuscular hemoglobin concentration (MCHC), were not examined. Third, this research used a single-center design with a small sample size; thus, the results could not be generalized to other ethnic groups. Thus, this research recommended additional studies encompassing extensive analyses involving detailed biochemical indicators, multi-center RCTs, and patients of different ethnicities to ensure better results.

## Conclusions

The findings of this study suggest a positive correlation between perioperative blood transfusion (PBT) and length of hospital stay (LOS) in patients with osteoporotic fractures. However, a nonlinear relationship between PBT and LOS was also observed, with a negative association between PBT and LOS when PBT levels were between 0 and 1.5 units and a positive association when PBT levels exceeded 1.5 units. It is recommended that PBT administration be carefully managed to reduce LOS and avoid associated complications and high hospitalization costs, with a maximum of 1.5 units indicated for osteoporotic fracture patients undergoing surgery. These findings have important implications for clinical practice, highlighting the need for healthcare providers to exercise caution while administering PBT and consider alternatives wherever possible. Future research could further explore the underlying factors contributing to the observed nonlinear relationship as well as the impact of different transfusion thresholds on patient outcomes in this population. Such studies may provide valuable insights into optimizing PBT administration in osteoporotic fracture patients and improve clinical decision-making. Future research may expand based on the present findings by exploring the factors contributing to this nonlinear relationship and investigating the impact of alternative transfusion thresholds on LOS in OFs patients. Such studies may provide valuable insights for clinicians and healthcare providers seeking to improve the management of OFs patients and improve their overall quality of care.

## Data Availability

The data that support the findings of this study are available from the corresponding author upon reasonable request.

## References

[1] Camacho PM (2020). AMERICAN ASSOCIATION OF CLINICAL ENDOCRINOLOGISTS/AMERICAN COLLEGE OF ENDOCRINOLOGY CLINICAL PRACTICE GUIDELINES FOR THE DIAGNOSIS AND TREATMENT OF POSTMENOPAUSAL OSTEOPOROSIS-2020 UPDATE. Endocr Pract.

[2] Almashrafi A (2016). Factors associated with prolonged length of stay following cardiac Surgery in a major referral hospital in Oman: a retrospective observational study. BMJ Open.

[3] *Practice guidelines for perioperative blood management: an updated report by the American Society of Anesthesiologists Task Force on Perioperative Blood Management**. Anesthesiology, 2015. 122(2): p. 241–75. 10.1097/ALN.0000000000000463 25545654

[4] Pazianas M, van der Geest S, Miller P (2014). Bisphosphonates and bone quality. Bonekey Rep.

[5] Mc Auley MT (2012). A whole-body mathematical model of cholesterol metabolism and its age-associated dysregulation. BMC Syst Biol.

[6] Veldurthy V (2016). Vitamin D, calcium homeostasis and aging. Bone Res.

[7] Tinmouth A (2006). Clinical consequences of red cell storage in the critically ill. Transfusion.

[8] Spahn DR (2010). Anemia and patient blood management in hip and knee Surgery: a systematic review of the literature. Anesthesiology.

[9] Vincent JL (2002). Anemia and blood transfusion in critically ill patients. JAMA.

[10] Brunskill SJ et al. *Red blood cell transfusion for people undergoing hip fracture Surgery*. Cochrane Database Syst Rev, 2015(4): p. Cd009699. 10.1002/14651858.CD009699.pub2 PMC11065123 25897628

[11] Lawrence VA (2003). Higher hb level is associated with better early functional recovery after hip fracture repair. Transfusion.

[12] Carless PA et al. *Transfusion thresholds and other strategies for guiding allogeneic red blood cell transfusion*. Cochrane Database Syst Rev, 2010(10): p. Cd002042. 10.1002/14651858.CD002042.pub2 20927728

[13] Adunsky A (2008). Discharge hemoglobin and functional outcome of elderly hip fractured patients undergoing rehabilitation. Eur J Phys Rehabil Med.

[14] Foss NB, Kristensen MT, Kehlet H (2008). Anaemia impedes functional mobility after hip fracture Surgery. Age Ageing.

[15] Bower WF (2010). Peri-operative blood transfusion increases length of hospital stay and number of postoperative Complications in non-cardiac surgical patients. Hong Kong Med J.

[16] Cai X et al. *Relationship between Preoperative Hemoglobin and Hospital Stays in Patients Receiving Prime Total Knee Arthroplasty* Comput Intell Neurosci, 2022. 2022: p. 3627688. 10.1155/2022/3627688 PMC9307341 35875776

